# Bioavailability of Thymol Incorporated into Gastro-Resistant Self-Emulsifying Pellets in Rabbits

**DOI:** 10.3390/ani15223238

**Published:** 2025-11-07

**Authors:** Radoslava Kristofova, Karin Zitterl-Eglseer, Fardad Firooznia, Andrea Laukova, Lubica Chrastinova, Monika Pogany Simonova, Margareta Takacsova, Kristina Bacova, Iveta Placha

**Affiliations:** 1 Department of Digestive Tract Physiology, Institute of Animal Physiology, Centre of Biosciences, Slovak Academy of Sciences, Soltesovej 4-6, 040 01 Kosice, Slovakia; kristofova@saske.sk (R.K.); laukova@saske.sk (A.L.); simonova@saske.sk (M.P.S.); takacsova@saske.sk (M.T.); bacovak@saske.sk (K.B.); 2 University of Veterinary Medicine and Pharmacy, Komenskeho 73, 040 01 Kosice, Slovakia; 3 Centre for Veterinary Systems Transformation and Sustainability, Clinical Department for Farm Animal and Food Systems Science, University of Veterinary Medicine, Veterinaerplatz 1, 1210 Vienna, Austria; karin.zitterl@vetmeduni.ac.at; 4 Department of Biology, The City College of New York, 160 Convent Avenue, New York, NY 10031, USA; ffirooznia@ccny.cuny.edu; 5 Animal Production Research Centre, National Agricultural and Food Centre, Hlohovecka 2, 951 41 Nitra-Luzianky, Slovakia

**Keywords:** thymol, self-emulsifying pellets, oral bioavailability, rabbits, metabolism

## Abstract

Thymol gastro-resistant self-emulsifying pellets were used to achieve thymol targeted release on the side of the intestine with the most intensive absorption to enhance its oral bioavailability. Forty-eight rabbits (35 d of age) were divided into two groups fed with a standard diet containing gastro-resistant enteric pellets (control, CG; without thymol, initial live weight 1350.0 ± 18.0, and experimental, EG; with thymol 250 mg/kg, initial live weight 1352.0 ± 19.9 g). The experiment lasted 28 days: thymol was administered for 21 days and then withdrawn for 7 days. Thymol was significantly higher in duodenal wall (DW) than in plasma during both periods (*p* = 0.0053, *p* < 0.0001). Significant correlation was established between thymol concentration in plasma and DW during its application (r_s_ = 0.9333, *p* < 0.001). Thymol was below the limit of quantitation in plasma, spleen and muscle only after its withdrawal, and its significantly higher concentration in kidney and fat than in plasma (*p* = 0.0182, *p* = 0.0003) and muscle (*p* = 0.0236, *p* = 0.0004) indicates its efficient accumulation. Thymol in gastro-resistant form prevented its degradation due to adverse conditions in the stomach and ensured its release at the site of greatest absorption in the small intestine.

## Simple Summary

In veterinary applications, thymol is of particular interest as a natural alternative to antibiotic growth promoters, contributing to gut health modulation, immune support and disease prevention. However, thymol’s clinical potential is significantly hindered by its poor aqueous solubility, which limits oral absorption and reduces systemic bioavailability. To overcome this limitation, we have focused our attention on thymol self-emulsifying pellets designed to improve its solubility and optimise release profiles. Thymol gastro-resistant self-emulsifying pellets effectively protected thymol from gastric degradation and enabled its targeted release and absorption in the small intestine. The stabilised thymol formulation promoted accumulation in tissue in rabbits during administration and persisted even after its withdrawal. The developed gastro-resistant thymol formulation represents a promising approach to enhance its oral bioavailability in a rabbit model.

## 1. Introduction

Oral administration remains a widely used route for drug delivery; however, it is inherently challenged by low and variable bioavailability, particularly for lipophilic compounds with poor aqueous solubility. Drugs administered orally must traverse the gastrointestinal tract, where factors such as solubility, degradation by digestive enzymes and first-pass metabolism substantially limit systemic exposure [1]. In this context, advanced formulation strategies are essential to overcome these obstacles and to improve therapeutic outcomes, and nanoparticles have emerged as a highly promising strategy to improve drug delivery [2].

Thymol, a monoterpene phenol predominantly found in *Thymus vulgaris* L., has been widely recognised for its broad spectrum of biological activities, including antimicrobial, antioxidant, anti-inflammatory and antiseptic properties. Due to its lipophilic nature, thymol’s therapeutic potential in medicine has attracted increasing scientific attention [3]. In veterinary applications, thymol is of particular interest as a natural alternative to antibiotic growth promoters, contributing to gut health modulation, immune support and disease prevention [4]. However, thymol’s clinical potential is significantly hindered by its poor aqueous solubility, which limits oral absorption and reduces systemic bioavailability.

Lipophilic compounds like thymol are primarily absorbed via the intestinal lymphatic system, a route that bypasses the hepatic first-pass metabolism and allows for higher systemic availability of active compounds [5]. Central to lymphatic transport are chylomicrons, large lipoprotein particles ranging from 80 to 1200 nm in diameter, which are synthesised in enterocytes in response to lipophilic molecules. Compared to smaller lipoproteins such as very low-density lipoprotein (30–80 nm), chylomicrons are more efficiently taken up by the intestinal lymphatic vessels, known as lacteals [6].

To overcome limitations of oral absorption, recent research has focused on the development of advanced formulation strategies designed to improve solubility and optimise drug release profiles. A notable example is the study by Macku et al. [7] who designed a self-emulsifying pellet formulation of thymol specifically tailored to the gastrointestinal tract of rabbits. Utilising a rational design approach guided by molecular-level structural characterisation and validated through ex vivo testing, the goal was to optimise the release profile and improve the absorption efficiency of thymol in a species-specific manner, taking into account the unique anatomical and physiological characteristics of the rabbit digestive system.

Accordingly, formulation strategies that promote the incorporation of lipophilic drugs like thymol into chylomicrons can significantly enhance lymphatic absorption. Lipid-based delivery systems, including self-emulsifying systems (SES), have proven effective in facilitating this process and improving the oral bioavailability of compounds with poor aqueous solubility [8].

To enhance the oral bioavailability of thymol and achieve a more efficient therapeutic effect, our study aimed to provide a detailed characterisation of its metabolic pathway in the rabbit organism following the dietary administration of thymol self-emulsifying pellets. We expect that our study will provide an answer to whether this form of thymol ensures more effective absorption in the small intestine as well as its greater accumulation in the tissues, which would subsequently result in a more effective impact on the health status of rabbits.

## 2. Materials and Methods

### 2.1. Experimental Diet

The standard diet consisted of a commercial diet for growing rabbits (KKZK, Liaharensky podnik Nitra a.s., Nitra, Slovakia), with ingredients and chemical composition as shown in Table 1. The feed was stored in the dark to protect against degradation processes. The Association of Official Analytical Chemists method [9] was used to determine the dry matter (no. 967.03, DM) in the diet, while DM amount was also determined for the tissues, gut content and faeces. Thymol (≥99.9%, Sigma-Aldrich, St. Louis, MO, USA) at a concentration of 250 mg/kg feed was incorporated into enteric pellets with thymol self-emulsifying systems (SES) developed by Macku et al. [7]. Briefly, the formulation process included thymol incorporation into SES matrices, followed by extrusion/spheronisation to form uniform pellets. These pellets were subsequently coated using a Wurster-type fluid bed apparatus to apply an enterosolvent coating. Specifically, an aqueous dispersion of the gastro-resistant polymer Eudragit^®^ L30 D55 (Evonik, Essen, Germany) was used to coat the pellets, effectively preventing premature thymol release under gastric conditions and enabling targeted release in the small intestine. The formulation was designed based on molecular-level characterisation to ensure efficient thymol solubilisation and release. Moreover, ex vivo testing demonstrated the capability of the SES to permeate the intestinal mucosa and deliver thymol directly to enterocytes, confirming the efficacy of the delivery system.

### 2.2. Thymol Stability in Feed

Thymol evaporation in feed was analysed every week during thymol application using high-performance liquid chromatography (HPLC) according to the modified method of Oceľová [10] and Pisarčíková et al. [11]. Samples were analysed in triplicate. Briefly, 2 mL of methanol was added to a glass tube containing 0.2 g of milled feed, and thymol was extracted in an ultrasonic bath. The methanolic extract was then analysed using the HPLC method with an Ultimate 3000 HPLC-system liquid chromatograph (Dionex, Sunnyvale, CA, USA). The chromatographic analyses were evaluated using Chromeleon^®^ Software Version 6.80 SR10 Build 2906 (Thermo Fisher Scientific, Waltham, MA, USA).

### 2.3. Animals, Experimental Design and Animal Care

A total of 48 rabbits of both sexes (an equal male-to-female ratio per treatment) at five weeks of age were randomly divided into two dietary treatment groups (24 rabbits in each); a control group (CG) fed a standard diet to which enteric pellets without thymol were added and an experimental group (EG) fed a standard diet into which enteric pellets with thymol (250 mg/kg) were incorporated. Plasma and tissue samples from the control group were used as blank samples to construct calibration curves. The rabbits of meat line M91, maternal albinotic line (cross breed New Zealand White, Bouscat rabbit, Argente Champagnet rabbit) and paternal acromalictic line (crossbreed Nitra’s rabbit, Californian rabbit, Big light silver) were used in this experiment. The animals were fed ad libitum and had free access to drinking water. All the experimental wire-net cages (61 cm × 34 cm × 33 cm) were kept in rooms with automatic temperature and humidity control through a digital thermograph (22 ± 4 °C, 70 ± 5%). A lighting regimen of 16 h light and 8 h dark was applied throughout the experiment. The experiment lasted 28 days. The EG rabbits received feed with thymol pellets for 21 days (56 d of age) and for the next 7 days (63 d of age) received pellets without thymol incorporated into feed.

### 2.4. Growth Performance

Body weight (BW) and feed intake (FI) were recorded individually once a week. The average daily FI, average daily weight gain (WG) and feed conversion ratio (FCR) were calculated at the end of the period (at 56 d of age and 63 d of age).

### 2.5. Animal Stunning, Slaughter, and Ethical Approval

This experiment was carried out at the experimental rabbit facility of the National Agricultural and Food Centre, Research Institute for Animal Production, Nitra, Slovakia.

Nine rabbits in each group were stunned at 56 or 63 d of age using electronarcosis (50 Hz, 0.3A/rabbit for 5 s), immediately hung by the hind legs on the processing line, and quickly bled by cutting the jugular veins and the carotid arteries. The operator performing exsanguination was trained in the technique and ensured precise placement of the blade high on the neck, ideally at the level of the first cervical vertebra. Blades used for decapitation were properly maintained and sufficiently sharp to achieve complete severance of the head with a single stroke. Rabbits were securely restrained to prevent any movement away from the blade, minimizing stress and ensuring accuracy [12]. This method of stunning followed by exsanguination was performed following the American Veterinary Medical Association [13], which recognizes electronarcosis followed by exsanguination as an acceptable and humane euthanasia method for rabbits when performed correctly.

### 2.6. Ethical Statement

All procedures involving animals were approved by the Institutional Ethics Committee and the State Veterinary and Food Administration of the Slovak Republic under protocol number SK U03021. Animals were handled by qualified personnel, and all efforts were made to minimize suffering and distress before stunning, ensuring that all procedures were conducted humanely and ethically. The authors confirm that they have followed EU standards for the protection of animals used for scientific purposes.

### 2.7. Sampling

To determine the thymol content in plasma, blood (1.5 mL) from nine rabbits was collected from the marginal ear vein (*vena auricularis*) into heparinised Eppendorf tubes, and plasma was obtained after centrifugation at 1180× *g* for 15 min. The gastrointestinal tract was removed from the body cavity and was divided into the small intestine (duodenum and jejunum), caecum and colon (n = 9). Caecum and colon contents were removed, and the small intestinal lumen was gently washed with a 0.9% NaCl solution. The obtained samples of duodenal wall (DW), jejunal wall (JW) and gut content together with plasma, liver, kidney, muscle (*longissimus thoracis et lumborum*, LTL) and spleen tissue, fat, and faeces were immediately frozen in liquid nitrogen and stored at −70 °C until analysed. All samples were collected on both experimental days (56 or 63 d of age).

### 2.8. Thymol Analyses in Plasma, Tissues, Large Intestinal Content and Faeces

Thymol was determined using solid-phase microextraction (SPME) coupled with gas chromatography/mass spectrometry (GC/MS). The analytical system comprised an Agilent 7890A gas chromatograph (Agilent Technologies, Wilmington, DE, USA) interfaced with an Agilent 5975C Mass Selective Detector (VL MSD with Triple-Axis Detector), Agilent Technologies, Wilmington, DE, USA). Chromatographic separation was achieved on an HP-5MS capillary column (Agilent 19091S-433) with dimensions of 30 m in length, 0.250 mm inner diameter and 0.25 μm film thickness. Helium served as the carrier gas under strictly controlled flow (an initial flow rate of 2.77 mL/min and a post-run flow of 1.3 mL/min) and inlet pressure (approximately 23.27 psi). Sample preparation was carried out using solid-phase microextraction (SPME), a solvent-free and efficient extraction technique that utilises the adsorption of analytes onto a polymer-coated fibre. For this purpose, a 65 μm polydimethylsiloxane/divinylbenzene (PDMS/DVB) StableFlex fibre assembly (23Ga, Supelco 57293-U, Sigma-Aldrich, St. Louis, MO, USA) was employed in combination with a PAL autosampler (CTC Analytics, Zwingen, Switzerland). Due to fibre wear, the SPME fibre was replaced after every 40 to 60 samples to ensure consistent extraction performance.

For determination of the total content of thymol plasma and homogenized tissue samples were enzymatically hydrolysed with β-glucuronidase from *Helix pomatia* (Sigma-Aldrich, St. Louis, MO, USA) at 37 °C and pH 5 for 30 min to cleave thymol conjugates. Following hydrolysis, samples were salted out with sodium chloride (1.00 g), acidified with phosphoric acid (85%) and spiked with an o-cresol internal standard. All reagent concentrations and incubation conditions were applied as described in the previous study by Oceľová et al. [14].

The prepared sample was transferred to the autosampler which initiated the extraction process with a pre-incubation phase lasting 10 s at 80 °C, followed by an active incubation with agitation at 500 rpm. This step ensured thorough mixing and consistent analyte partitioning. The fibre was then inserted into the headspace of a sealed sample vial through a polytetrafluoroethylene/butyl rubber septum, where the analytes were allowed to equilibrate and adsorb onto the fibre for 35 min at 80 °C. Partitioning occurred between the aqueous sample matrix and its headspace according to the physicochemical properties and partition coefficients of the compounds. Following adsorption, the fibre was retracted and immediately transferred to the GC injector for thermal desorption at 250 °C for 5 min under split injection conditions (split ratio 1:1) by the autosampler. This step enabled the release of the analytes into the gas chromatographic column for subsequent separation and detection. The GC oven program began at 60 °C with an initial hold of 5 min, ramped at 4 °C/min to 120 °C, and subsequently increased at 20 °C/min to 240 °C. A transfer line maintained at 280 °C ensured efficient delivery of the analytes into the mass spectrometer. Mass spectrometric detection was conducted in Selected Ion Monitoring (SIM) mode to improve sensitivity and specificity. Data acquisition was organised in three SIM groups: Group 1 (starting at 5 min) monitored key hydrocarbon ions (*m*/*z* 57.00, 184.00, 198.00, 212.00, and 226.00) at a dwell time of 30 ms per ion; Group 2 (commencing at 7 min) focused on o-cresol (the internal standard) by monitoring ions at *m*/*z* 107.00 and 108.00 with dwell times of 150 ms each; and Group 3 (initiated at 14 min) was dedicated to thymol, capturing ions at *m*/*z* 135.00 and 150.00 with dwell times of 150 ms each. The MS source was maintained at 230−250 °C and the quadrupole at 150 °C (up to 200 °C) for stable and accurate mass detection.

Quantification was achieved using calibration curves generated by plotting the peak-area ratios of thymol to o-cresol (internal standard; Sigma-Aldrich, St. Louis, MO, USA) against known thymol concentrations. The limit of quantification (LOQ) represents the lowest concentration of thymol that can be quantified with acceptable accuracy and precision under the validated analytical conditions. In some samples, automatic peak integration by the software was not possible due to low signal intensity and background noise. Therefore, manual peak integration was performed to estimate thymol concentrations in these cases. These manually integrated values are considered to be below the LOQ but above the limit of detection (LOD). Calibration curves were prepared from blank samples (CG) of plasma and tissues spiked with thymol (Applichem, Darmstadt, Germany) in concentrations 50, 100, 200, 400, 800, 1000 and 2000 ng/mL for plasma and the same concentrations in ng/g for tissues such as the kidney, DW, JW, caecum and colon content as well as faecal samples. In the case of liver tissue, the calibration range included 6.25, 25, 50, 100, 250, 400 and 800 ng/g thymol and for muscle an additional concentration of 2.0 ng/g. For spleen samples calibration points from 2.0 to 250 ng/g whereas in fat samples from 1.0 to 100 ng/g were used. The retention time of thymol was consistently around 18 min, while the o-cresol peak appeared around 9 min in all samples.

### 2.9. Statistical Analysis

The statistical analyses were performed using GraphPad Prism version 10.4.2 for Windows, GraphPad Software. The Kolmogorov–Smirnov test was used to assess whether the data met the assumption of normality required for parametric statistical analyses. When the data did not meet the assumption of normal distribution, non-parametric tests were applied. The Kruskal–Wallis test with post hoc Dunn’s Multiple Comparison test was used to determine the differences between plasma, DW, JW, colon and caecum; DW, JW, colon, caecum and faeces; and plasma, kidney, fat, spleen, liver and muscle. This ensured that the statistical approach remained appropriate despite deviations from parametric assumptions. The results are presented as mean value ± standard error of mean (SEM). Differences were considered significant at *p* < 0.05. Correlations of thymol concentrations between plasma and DW, plasma and kidney, plasma and muscle, DW and kidney, DW and muscle and JW and muscle were analysed using nonparametric Spearman’s Rank Correlation and expressed as Spearman’s correlation coefficient (r_s_). The experimental unit was the cage of animals.

## 3. Results

### 3.1. Growth Performance

Animals were in good health, and growth performance of the broiler rabbits was not influenced by thymol addition. Initial live weight was 1350.0 ± 18.0 g in the CG and 1352.0 ± 19.9 g in the EG (2480 ± 19.3 g in the CG, 2497 ± 46.9 g in the EG at 56 d of age, and 2528 ± 12.3 g in the CG, 2525 ± 63.2 g in the EG at 63 d of age). The average weight gain per rabbit was 34.17 ± 1.24 g/day in CG, 36.25 ± 1.48 g/day in EG at 56 d of age, and 42.57 ± 5.22 g/day in CG, 40.24 ± 2.94 g/day in EG at 63 d of age. The conversion ratio was 4.35 ± 0.22 in CG, 4.44 ± 0.25 in EG at 56 d of age, and 3.83 ± 0.28 in CG, 4.24 ± 0.29 in EG at 63 d of age.

### 3.2. Thymol Stability in Feed

The concentration of thymol in feed during the period with thymol application was relatively stable, 246.1 ± 5.8 µg/g DM-0 d, 197.8 ± 8.3 µg/g DM-7 d, 221.9 ± 5.1 µg/g DM-14 d.

### 3.3. Comparison of Thymol Concentration Between Plasma, Duodenal and Jejunal Wall, Colon and Caecum Content to Obtain a View on Thymol Absorption from the Intestinal Tract

**Figure 1:**
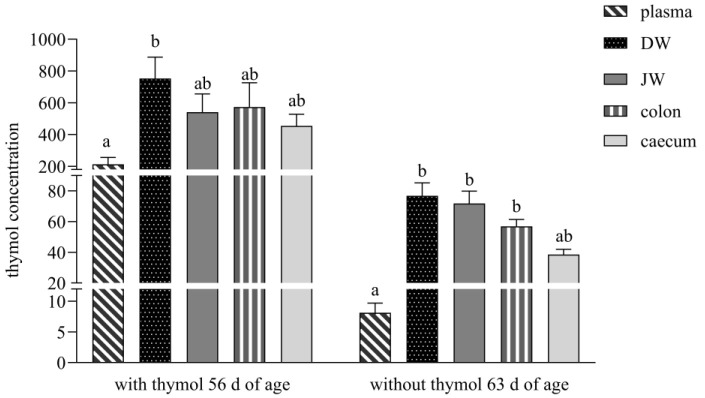
Thymol content in plasma (ng/mL), duodenal wall-DW (ng/g dry matter-DM), jejunal wall-JW (ng/g DM), colon (ng/g DM) and caecum content (ng/g DM). Different superscript letters identify significant differences (*p* < 0.05). Data are presented as mean value ± standard error of mean (SEM).

During both periods, thymol was above the limit of quantitation (LOQ), except in plasma after thymol withdrawal was below LOQ. Thymol in DW was significantly higher than in plasma during both periods (21 d with thymol *p* = 0.0053, 7 d without thymol *p* < 0.0001) and in JW (*p* = 0.0001) and colon (*p* = 0.0038) at d 7 without thymol. Thymol levels were not strongly divergent in all parts of the intestinal tract during both periods and can therefore be regarded as “in balance” (Figure 1).

### 3.4. Comparison of Thymol Concentration Between Duodenal and Jejunal Wall, Colon and Caecum Content and Faeces to Obtain View Tn Thymol Passage Through the Intestinal Tract

**Figure 2:**
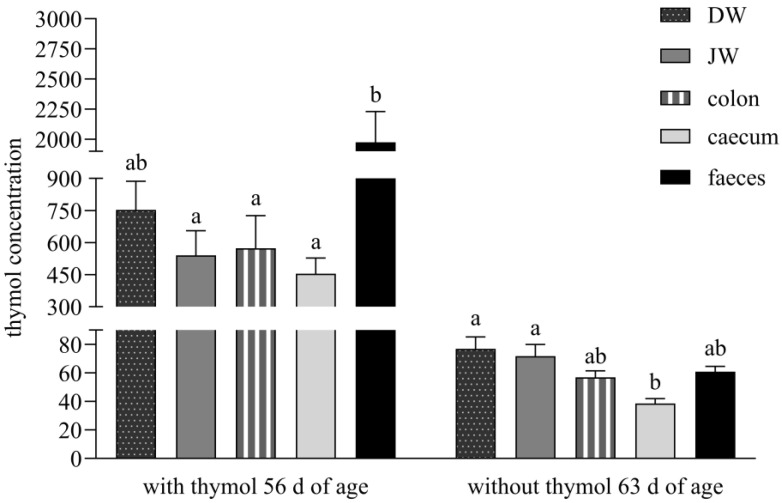
Thymol content in duodenal wall-DW (ng/g dry matter-DM), jejunal wall-JW (ng/g DM), colon content (ng/g DM), caecum content (ng/g DM) and faeces (ng/g DM). Different superscript letters identify significant differences (*p* < 0.05). Data are presented as mean value ± standard error of mean (SEM).

During the period with thymol application its concentration in faeces was significantly higher than in JW (*p* = 0.0033), colon (*p* = 0.0022) and caecum (*p* = 0.0011); during the period without thymol administration, thymol concentrations were significantly lower in caecum than in DW (*p* = 0.0023) and JW (*p* = 0.0132, Figure 2).

### 3.5. Comparison of Thymol Concentration in Plasma, Liver, Kidney, Spleen, Fat and Muscle to Obtain a View on Thymol Distribution and Accumulation

**Figure 3:**
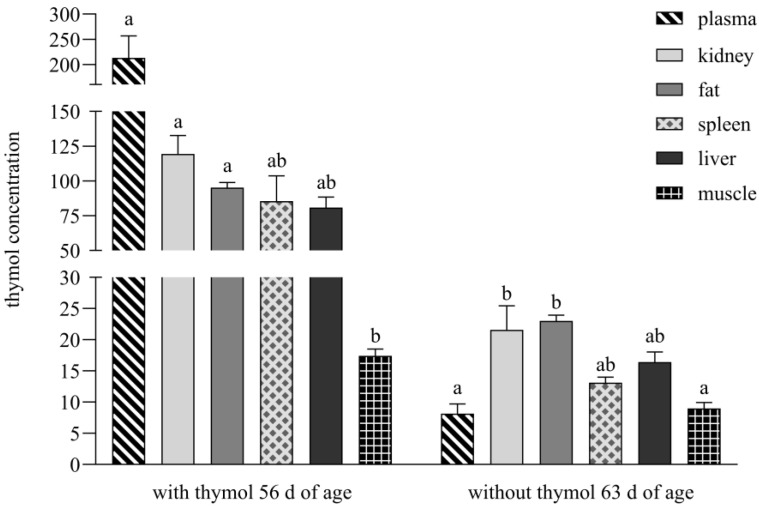
Thymol content in plasma (ng/mL), liver (ng/g DM), kidney (ng/g DM), spleen (ng/g DM), fat (ng/g DM) and muscle (ng/g DM). Different superscript letters identify significant differences (*p* < 0.05). Data are presented as mean value ± standard error of mean (SEM).

During both periods, thymol was above LOQ in all tissues; after thymol withdrawal, it was below LOQ only in plasma, spleen and muscle. During thymol application its concentration in plasma (*p* < 0.0001), kidney (*p* = 0.0002) and fat (*p* = 0.0095) was significantly higher than in muscle; after withdrawal, it was significantly higher in kidney and fat than in plasma (*p* = 0.0182, *p* = 0.0003) and muscle (*p* = 0.0236, *p* = 0.0004; Figure 3).

### 3.6. Correlation Between Thymol Concentration in Plasma and Duodenal Wall, Plasma and Kidney, Duodenal Wall and Kidney, Plasma and Muscle, Duodenal Wall and Muscle, and Jejunal Wall and Muscle

**Figure 4:**
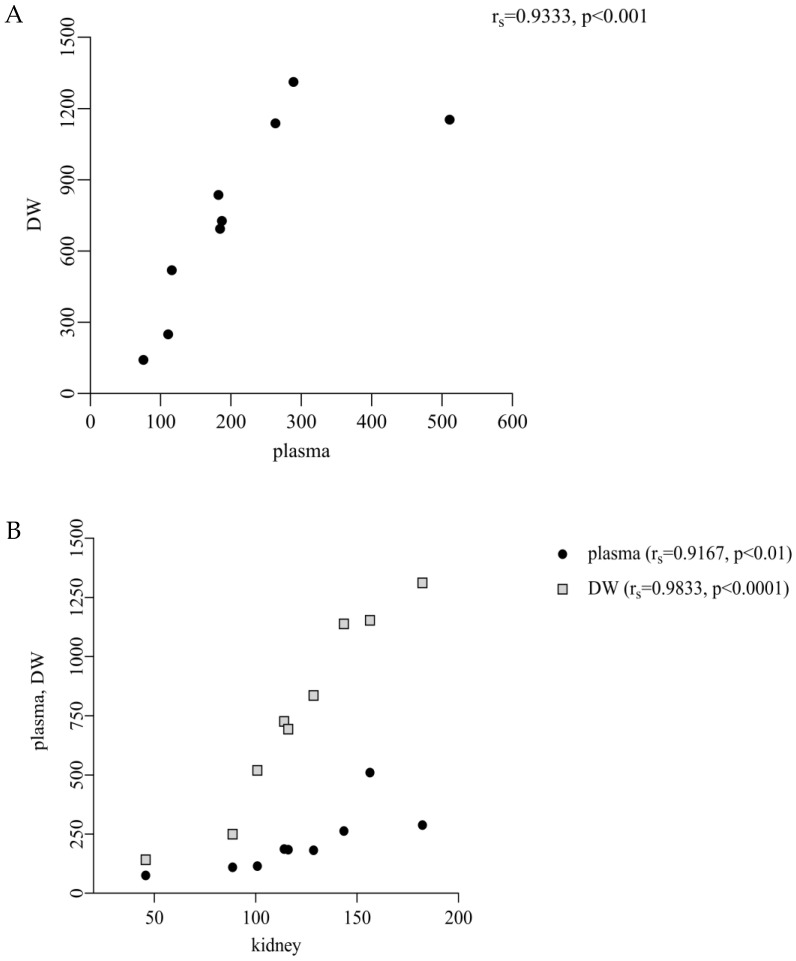

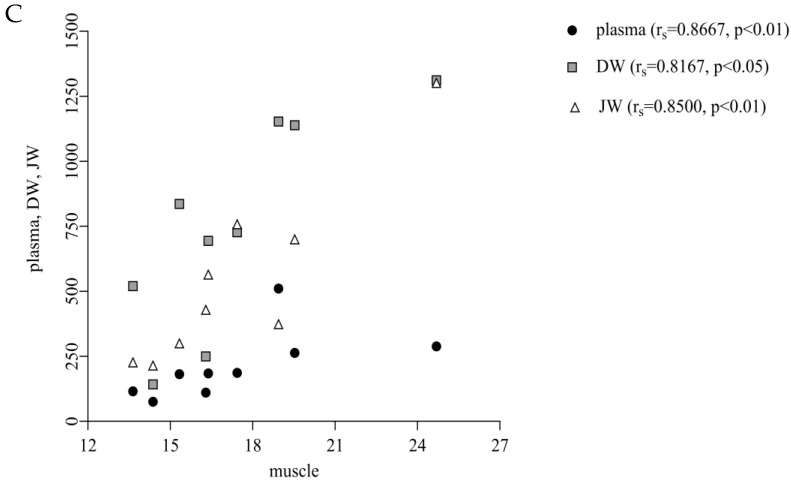
(**A**–**C**)**.** Nonparametric Spearmen correlation (r_s_) between thymol concentration in plasma (ng/mL) and duodenal wall-DW (ng/g dry matter-DM, (**A**)); plasma (ng/mL) and kidney (ng/g DM, (**B**)); DW (ng/g DM) and kidney (ng/g DM, (**B**)); plasma (ng/mL) and muscle (ng/g DM, (**C**)); DW and muscle (ng/g DM, (**C**)); jejunal wall-JW (ng/g DM) and muscle (ng/g DM, (**C**)).

A significant correlation was established between thymol concentration in plasma and DW (r_s_ = 0.9333, *p* < 0.001; Figure 4A), plasma and kidney (r_s_ = 0.9167, *p* < 0.01; Figure 4B), DW and kidney (r_s_ = 0.9833, *p* < 0.0001; Figure 4B), plasma and muscle (r_s_ = 0.8667, *p* < 0.01; Figure 4C), DW and muscle (r_s_ = 0.8167, *p* < 0.05; Figure 4C), and JW and muscle (r_s_ = 0.8500, *p* < 0.01; Figure 4C).

## 4. Discussion

Plant-derived bioactive compounds which are applied into animal feed can be degraded by adverse stomach conditions or rapidly absorbed in the upper GIT. These circumstances cause their inability to reach the lower parts of the intestinal tract, where more pathogens reside and propagate, and in this way reduce the effectiveness of phytoadditives [15,16].

Macku et al. [7] in their study developed pellets with a thymol self-emulsifying system (SES), and in an ex vivo study, they confirmed the transport of thymol through the mucus layer of the rabbit small intestine and its delivery to enterocytes. The thymol SES used in our study ensured a relatively balanced concentration of thymol in the GIT during the period with thymol addition, with no significance found between individual parts of intestine. Thymol concentration in DW was 1.4-, 1.3- and 1.7-times higher than in JW, colon and caecum (Figure 1). In contrast, Bacova et al. [17], in their study with the addition of the same concentration of thymol in a powder non-pelleted form, found the concentration of thymol to be 2.5- and 3.3-times lower in DW in comparison with the colon and caecum. They explained this finding by the fast passage of thymol to the colon, with subsequent excretion, as a consequence of the inability to cross the membranes of the intestinal wall by the time it reached the colon. We assume that in our study, only a low percentage of thymol was released from pelleted particles in the stomach, and a larger quantity of thymol was slowly released in the small intestine.

Thymol, as a lipophilic substance, is converted during biotransformation to hydrophilic metabolites, mainly thymol sulphate and glucuronide, which are not only excreted from the organism but are also converted back to the parent compound thymol by metabolic processes of enzymes located in the intestinal wall and microflora [17]. Briefly, during enteric recycling some percentage of absorbed parent drugs are metabolised in intestinal cells: some are effluxed back into the lumen and some are transported to the blood. This process is repeated again and again, until the compound is eliminated from the body [18]. We can hypothesise that the release of larger amounts of thymol in the small intestine together with its intensive biotransformation processes in the intestinal wall led to equilibrium through the different parts of the small intestine.

Unlike in the study Bacova et al. [17], who with the addition of thymol in powder form detected thymol during the withdrawn period only under the detection limit in all intestinal segments, the thymol concentration in our study during this period was still above the limit of quantitation and in balance in all parts of the intestine (in DW 1.1-, 1.3- and 1.9-times higher than in JW, colon and caecum). The obtained results showed that thymol and its metabolites systematically circulated in the rabbit organism even after its withdrawal. Placha et al. [19] showed in their study that the metabolic processes of thymol were more active as a consequence of caecotrophy, which ensured thymol reingestion and reabsorption. We assume that the slow release of thymol from pellets in the small intestine and its intensive repeated biotransformation processes, together with processes of caecotrophy, caused its high concentration even after its withdrawal.

We found a significantly lower concentration of thymol in plasma in comparison with DW and a significant correlation between them (213.1 ng/mL vs. 752.7 ng/g DM, *p* < 0.05; r_s_ = 0.9333, *p* < 0.001; Figure 1 and Figure 4A), which revealed its intensive absorption in DW. These results are comparable with the previous studies of Bacova et al. [17], who applied thymol in powder form into rabbit diets, and Oceľová et al. [14] with application of thyme essential oil in poultry diets. These authors pointed to the efficient absorption of thymol from the gut content into the duodenal wall. In our study, the amount of thymol in plasma after its withdrawal was below the limit of quantitation, which indicated that thymol was no longer being ingested through feed. Thymol was detected above the limit during the period without thymol addition in all intestinal segments, and the highest concentrations in DW and JW indicated continuous biotransformation processes also during this period (Figure 1).

During administration, the significantly higher concentration of thymol in faeces than in all intestinal segments except DW indicates sustained release of thymol throughout the lower gastrointestinal tract. We hypothesise that gastro-resistant pellets achieved targeted release and absorption in the duodenum, maximising thymol bioavailability at its most effective absorption site [7]. Following the withdrawal period, the significantly lower thymol concentration in the caecum versus DW and JW supports the hypothesis that reingested caecotrophs release thymol anew in the small intestine, contributing to secondary absorption (Figure 2).

The stomach is not a major absorption organ, and absorption is limited by its small epithelial surface which has a larger number of mucosal than absorptive cells. However, small non-ionised lipophilic molecules like thymol, with a dissociation constant pKA higher than 7.5 (pKA = 10.62, weak acid), are capable of being absorbed in the stomach [10,18]. To improve their efficacy means that only minimal amounts can be released in the stomach, and high amounts need to be delivered to the small intestine, where they are released mainly before excretion from the organism [15]. The balance between thymol content in all parts of the small intestine and its relatively high concentration in comparison with faeces during both periods in our experiment, indicated its low absorption in the stomach and its delivery in high amounts to the absorption side of the intestine where intensive absorption occurred (Figure 2).

During the period with thymol addition, its concentrations were above LOQ in the entire intestinal tract as well as examined tissues and organs, but during the period without supplementation, its concentrations were below LOQ in plasma, spleen and muscle (Figure 3). A completely different situation was recorded in the study of Bacova et al. [17], who applied thymol in powder form into rabbit diets. They analysed thymol as being below the detection limit in muscle and fat tissues as early as during its application, and after thymol withdrawal, amount of thymol was below the detection limit in all intestinal segments and organs and was not detected in the spleen.

The oral administration of compounds often presents challenges in achieving optimal bioavailability due to various physiological barriers, including enzymatic degradation in the gastrointestinal tract, limited absorption in the small intestine and extensive first-pass hepatic metabolism. The route of absorption for orally administered compounds is largely determined by their molecular weight and solubility, with compounds being transported either via the portal vein or the intestinal lymphatic system [5].

In the present study, a lipid-based pellet formulation of thymol, utilising a self-emulsifying system (SES), demonstrated superior bioavailability and prolonged tissue retention compared to its powdered counterpart. This formulation strategy appears to enhance solubility and provide protection against gastric degradation. In contrast, the powdered form of thymol, as employed in the study by Bacova et al. [17], exhibited limited solubility and increased susceptibility to degradation.

The observed differences in tissue distribution and retention may be attributed to enhanced intestinal lymphatic absorption facilitated by the lipid-based formulation. Lipophilic compounds such as thymol, when incorporated into lipid-based systems like SES, can be taken up by enterocytes and integrated into chylomicrons. These chylomicrons subsequently enter the intestinal lymphatic system rather than the portal blood, effectively bypassing hepatic first-pass metabolism and allowing a larger proportion of the intact compound to reach systemic circulation [6].

This mechanism of absorption appears to result in more efficient distribution of thymol to the peripheral tissues, particularly adipose tissue, where it accumulates and forms a depot that slowly releases the compound after its withdrawal. This depot effect may explain the persistent detectability of thymol in the liver, kidneys and fat, even after its withdrawal, suggesting redistribution from lipid stores, a prolonged pharmacokinetic profile and delayed elimination [20].

A significant positive correlation between thymol concentrations in plasma and kidney (r_s_ = 0.9167, *p* < 0.01), as well as between DW and renal levels (r_s_ = 0.9833, *p* < 0.0001; Figure 4B), suggests that renal accumulation of thymol is closely dependent on the extent of its systemic absorption. As the kidney represents a primary route for eliminating polar metabolites, the observed correlations support the hypothesis that increased gastrointestinal absorption leads to enhanced systemic availability and subsequent renal exposure. This aligns with established pharmacokinetic principles, according to which lipophilic compounds such as thymol undergo hepatic metabolism to form more polar, water-soluble metabolites that are then preferentially excreted via the kidneys [21]. In this context, we suggest that thymol undergoes metabolic conversion into hydrophilic metabolites, which are subsequently cleared via the renal route, thereby explaining the strong correlation between the plasma and kidney concentrations observed in our study. Similarly, the positive correlations between plasma and muscle (r_s_ = 0.8667, *p* < 0.01) and DW, JW and muscle (r_s_ = 0.8167-DW, *p* < 0.05; r_s_ = 0.8500-JW, *p* < 0.01; Figure 4C) indicate that muscle uptake follows intestinal absorption. We suggest that once absorbed, thymol is incorporated into chylomicrons and transported via the lymphatic system to peripheral tissues, including muscle, resulting in muscle deposition that directly reflects the extent of intestinal uptake [5].

In contrast, following the withdrawal period, thymol levels below LOQ were observed in the spleen, plasma or muscle following administration of the pellet formulation (Figure 3). These tissues are subject to rapid clearance (plasma) or likely represent sites with minimal capacity for accumulation [5]. Notably, when thymol was administered in powdered form, it was entirely undetectable in the spleen during the withdrawal period and remained below the detection threshold in all other tissues [17].

The variations in tissue distribution and prolonged persistence of thymol can thus be attributed to the distinct pharmaceutical formulations and the corresponding absorption mechanisms. The stabilised formulation likely activates the lymphatic route, leading to enhanced absorption, reduced first-pass metabolism and increased retention in target tissues, which was confirmed by thymol detection above the limit in all examined tissues during application. These findings suggest that incorporating thymol into lipid-based carriers facilitates its integration into chylomicrons, thereby enhancing lymphatic transport and systemic bioavailability. This mechanism may underpin the observed differences in tissue distribution between the pellet and powdered formulations, with the pellet form demonstrating significantly higher accumulation in target tissues due to more efficient lymphatic uptake.

## 5. Conclusions

The gastro-resistant self-emulsifying thymol pellets developed in this study successfully achieved targeted duodenal release and absorption of thymol, effectively bypassing extensive hepatic first-pass metabolism. By facilitating chylomicron-mediated lymphatic transport, these pellets enabled systemic redistribution of thymol, resulting in notable accumulation not only in the intestinal wall but also in adipose and muscle tissues. The combination of thymol’s intrinsic lipophilicity and sustained lymphatic delivery led to the establishment of secondary tissue depots, thereby prolonging systemic exposure. These findings suggest that stabilised thymol formulations represent a promising strategy to enhance its oral bioavailability in rabbits.

## Figures and Tables

**Table 1:** Ingredients and chemical composition (g/kg DM) of the experimental diet.

Ingredients	g/kg DM	Composition	g/kg DM
Dehydrated lucerne meal	337.33	Dry matter (g/kg)	937.03
Dry malting sprouts	140.56	Organic compounds	779.42
Oats	121.81	Nitrogen free extract	416.32
Wheat bran	84.33	Acid detergent fibre	194.99
Barley	74.96	Crude fibre	166.60
Extracted sunflower meal	51.54	Crude protein	165.48
Extracted rapeseed meal	51.54	Cellulose	152.83
Dried distiller grains with solubles	46.85	Hemicellulose	135.68
Premix ^1^	15.93	Starch	124.72
Limestone	9.37	Ash	64.84
Sodium chloride	2.81	Fat	31.02
Total	937.03	Metabolic energy, MJ/kg	9.91

^1^ The vitamin-mineral premix provided per kg of complete diet: Retinyl acetate 5.16 mg, Cholecalciferol 0.03 mg, Tocopherol 0.03 mg, Thiamine 0.8 mg, Riboflavin 3.0 mg, Pyridoxine 2.0 mg, Cyanocobalamin 0.02 mg, Niacin 38 mg, Folic acid 0.6 mg, Calcium 1.8 mg, Iron 70 mg, Zinc 66 mg, Copper 15 mg, Selenium 0.25 mg. DM: dry matter.

## Data Availability

The data that support the findings of this study are available upon request from the corresponding author.
